# Robust benchmarking of DeepLabv3 hybrid models for multiclass fundus screening and referable-disease triage on the FIVES dataset

**DOI:** 10.3389/fmed.2026.1771083

**Published:** 2026-03-31

**Authors:** Igor Garcia-Atutxa, Laura Martinez-Baliu, Francisca Villanueva-Flores

**Affiliations:** 1 Escuela Politécnica Superior, Universidad Católica de Murcia (UCAM), Murcia, Spain; 2 Universidad Tecmilenio, Azcapotzalco, Mexico; 3 Universitat Oberta de Catalunya, Barcelona, Spain; 4 Centro de Investigación en Ciencia Aplicada y Tecnología Avanzada, Unidad Morelos del Instituto Politécnico Nacional, Xochitepec, Mexico

**Keywords:** age-related macular degeneration, DeepLabv3, diabetic retinopathy, fundus photography, glaucoma, referable-disease triage

## Abstract

**Background:**

Automated analysis of color fundus photographs can support scalable screening for diabetic retinopathy (DR), age-related macular degeneration (AMD), and glaucoma, but single-run reporting and accuracy-only summaries can mask clinically relevant instabilities and failure modes.

**Methods:**

Using the public FIVES dataset, we benchmarked six deep learning configurations for four-class fundus screening (AMD, DR, glaucoma, normal): three DeepLabv3–backbone hybrids (ResNet50, DenseNet121, EfficientNet-B0) and three backbone-only classifiers. All experiments were evaluated using five independent stratified splits generated with different random seeds (*n* = 5 runs), each defining a distinct 20% held-out test set. Models were trained on the remaining 80% (training/validation), and all reported metrics are computed on the 20% test set of each run and summarized as mean ± SD across runs. Performance was summarized with accuracy, sensitivity, specificity, and one-vs.-rest AUC; we further characterized clinical behavior via row-normalized confusion matrices, per-class precision/recall/F1, and a screening-style binary triage setting (referable = AMD ∪ DR ∪ glaucoma vs. normal).

**Results:**

Hybrid models consistently achieved higher discrimination than simple classifiers (AUC 0.969–0.979 vs. 0.908–0.920), despite similar accuracies (0.924–0.941). The selected model, DeepLabv3–DenseNet121, reached the highest AUC (0.979 ± 0.009). Class-wise analysis revealed strong performance for Normal (F1 0.970 ± 0.014) and Glaucoma (F1 0.896 ± 0.048), while DR was the main bottleneck (sensitivity 0.738 ± 0.117), with most DR errors redistributed to AMD (13.6%) and Glaucoma (12.0%) and minimal confusion with Normal (0.5%). In binary triage, the model achieved sensitivity 0.993 ± 0.011 and specificity 0.963 ± 0.034, with PPV 0.987 ± 0.013 and NPV 0.980 ± 0.032, and a stable referral rate (∼0.73–0.77) across runs.

**Conclusion:**

DeepLabv3-based hybrids provide a robust advantage in AUC for multiclass fundus screening on FIVES. The residual risk concentrates in the DR–AMD–Glaucoma decision boundary, suggesting that deployment-oriented policies should prioritize conservative handling of DR-adjacent cases while leveraging the stability of Normal predictions for screening workflows.

## 1 Introduction

Vision impairment is a growing global health issue. The World Health Organization estimates that at least 2,200 million people live with some form of visual impairment, with half of these cases potentially preventable or yet to be addressed (1). Retinal diseases, including diabetic retinopathy (DR), age-related macular degeneration (AMD), and glaucoma, are leading causes of irreversible vision loss worldwide (2–4). DR affects 20–30% of individuals with diabetes, contributing to over a million cases of blindness (5, 6). AMD is expected to affect hundreds of millions in the coming decades, while glaucoma remains a leading cause of irreversible blindness despite available treatments (7, 8).

Color fundus photography is central to screening and monitoring these conditions, enabling detection of microaneurysms, hemorrhages, exudates, and optic disc cupping. However, manual interpretation is time-consuming, subject to inter-observer variability, and difficult to scale in settings with limited specialist access. Scalable automated tools can support teleophthalmology and technician-operated screening by improving triage efficiency and prioritizing high-risk cases (9–12).

Deep learning, particularly convolutional neural networks (CNNs), has transformed fundus image analysis. CNNs trained on large datasets can detect referable DR with expert-level performance, catalyzing clinical AI systems (13), and subsequent work extended deep models to AMD and glaucoma screening and grading tasks (14–17). More recently, hybrid paradigms combining CNN feature extraction with transformer-style global context modeling have gained traction across medical imaging, motivating renewed benchmarking of model families under consistent protocols and robustness checks (18, 19). At the same time, the field has progressed from single-disease detection toward multi-disease screening, where algorithms must distinguish multiple pathologies within clinically constrained workflows (20–22). In such settings, practical screening often benefits from a small set of actionable categories (e.g., DR vs. AMD vs. glaucoma vs. normal), and evaluation should capture not only average discrimination but also class-wise error structure and operational behavior under screening-like decisions (e.g., “referable” vs. “normal”).

Although originally developed for semantic segmentation, DeepLabv3 provides multi-scale context aggregation via atrous convolutions, which can be advantageous for learning discriminative fundus representations. Importantly, our pipeline is not a classification-via-segmentation framework: we do not predict anatomical/lesion masks and then use those predicted masks to drive the final disease decision. Instead, we use segmentation-style architecture purely as a feature-learning backbone and train it end-to-end for image-level classification by replacing the segmentation head with a four-class classification head. When a mask is used in our workflow, it is only a preprocessing aid to define the retinal ROI and suppress background. Here, we benchmark DeepLabv3–backbone hybrids against matched backbone-only classifiers for multiclass screening and a referable-vs-normal triage scenario (23, 24).

This work benchmarks six configurations on the public FIVES dataset (25) for four-class fundus screening (AMD, DR, glaucoma, normal): three DeepLabv3–backbone hybrids and three matched backbone-only classifiers. DeepLabv3 is repurposed as an image-level classifier via ASPP-based multi-scale context aggregation, enabling a backbone-matched comparison under a unified training pipeline. We report accuracy, sensitivity, specificity, and OvR-AUC, and characterize failure modes via row-normalized confusion matrices, per-class metrics, and a screening-style binary triage setting (referable = AMD ∪ DR ∪ glaucoma vs. normal). The scientific question is whether segmentation-style multi-scale context aggregation yields a consistent discrimination advantage over backbone-only classifiers in this setting; findings should be interpreted as within-dataset architecture effects that require external validation for clinical claims.

## 2 Materials and methods

### 2.1 Study design

This work was designed as a retrospective benchmarking study evaluating deep learning models for four-class disease recognition from color fundus photographs (AMD, DR, glaucoma, normal). All models were trained under a consistent protocol and evaluated using five independent seed-controlled splits (*n* = 5 runs) to quantify robustness. The primary hypothesis is that incorporating DeepLabv3’s ASPP-based multi-scale context aggregation (repurposed for classification) improves discrimination relative to backbone-only classifiers under the same training conditions, and that any gains persist across independent test splits.

### 2.2 Dataset and imaging protocol

**FIGURE 1:**
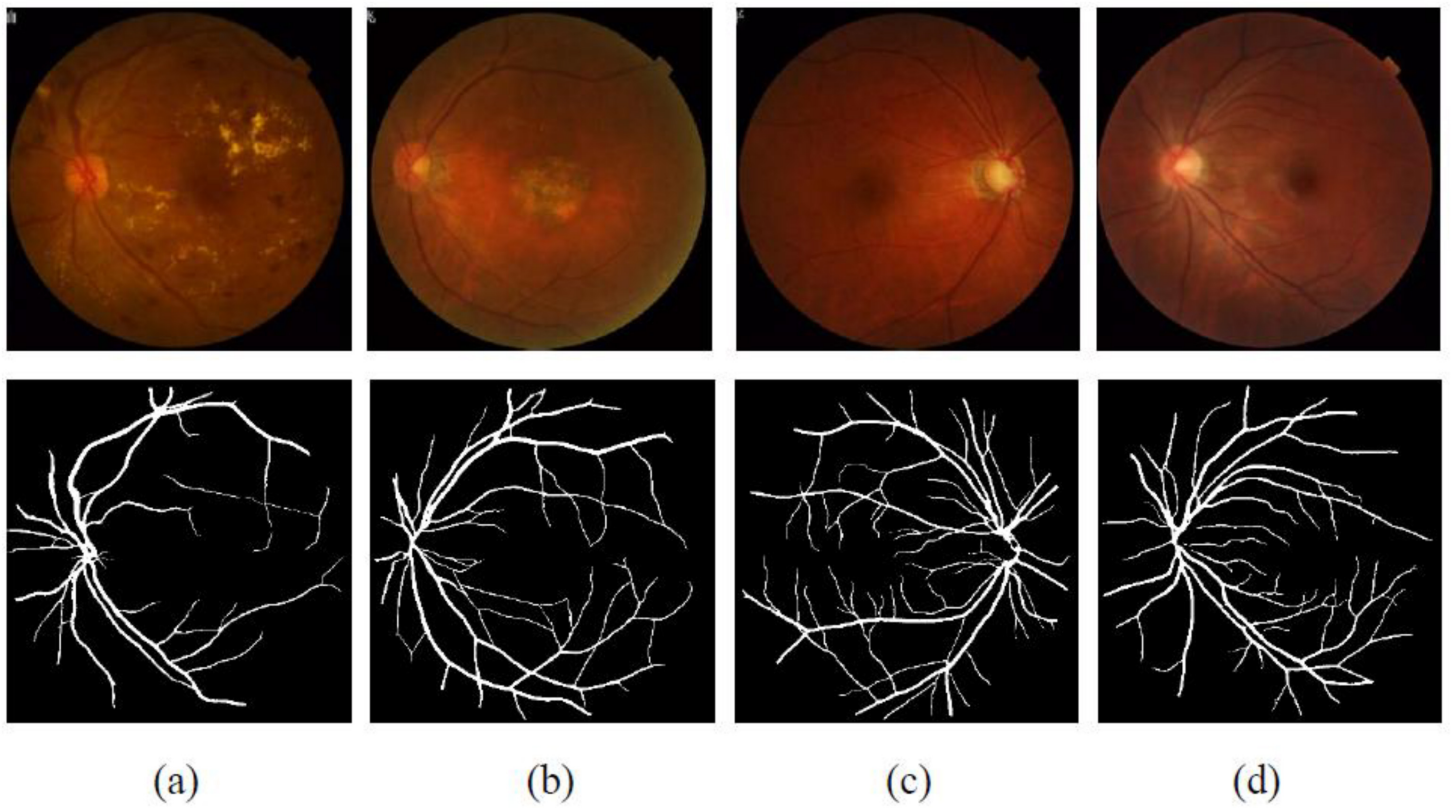
Illustrations of fundus images with their corresponding retinal vascular segmentation provided by FIVES: **(a)** diabetic retinopathy (DR), **(b)** age-related macular degeneration (AMD), **(c)** glaucoma, and **(d)** healthy subjects.

Experiments were conducted using the public FIVES (Fundus Image Vessel Segmentation) dataset, which provides color fundus photographs and corresponding pixel-wise vessel annotations. FIVES also provides image-level diagnostic labels (DR, AMD, glaucoma, normal), which are the targets used for our four-class classification task. The provided vessel masks are used only to define the retinal ROI during preprocessing and are not used as supervised segmentation targets in our experiments. The dataset includes 800 images acquired between 2016 and 2021 from 573 subjects spanning a broad age range (Figure 1). Images were collected using a TRC-NW8 fundus camera with a 50° field of view centered on the macula. Pharmacological pupil dilation was performed prior to imaging, and a small proportion of images with reduced legibility was retained to reflect realistic acquisition conditions. The original dataset collection reported informed consent and ethics approval; in this work we used the publicly released, de-identified data and adhered to the dataset’s stated usage conditions (25).

FIVES is class-balanced, containing 800 images with 200 images per category (AMD, DR, glaucoma, and normal). Table 1 summarizes the dataset composition used in this study. Importantly, the public FIVES release provides disease category labels at the image level and does not include within-disease severity staging (e.g., mild/moderate/severe/PDR for DR or staged glaucoma). Therefore, our classification task is defined at the four-category disease level, and stage-specific class imbalance cannot be assessed within FIVES. Note that while the multiclass setting is balanced (200 per class), the screening-oriented “referable vs. normal” triage is inherently imbalanced (referable = AMD+DR+glaucoma = 600 vs. normal = 200).

**TABLE 1:** FIVES dataset composition and label granularity used in this study.

Label (class)	Images (n)	Proportion (%)	Severity/stage labels available in FIVES
AMD	200	25.0	No (single image-level disease category)
DR	200	25.0	No (single image-level disease category)
Glaucoma	200	25.0	No (single image-level disease category)
Normal	200	25.0	N/A
Total	800	100	–

### 2.3 Preprocessing

To ensure architectural compatibility and standardize the input distribution across models, all fundus images were resized to 224 × 224 pixels. Intensity normalization was applied prior to training to stabilize optimization and reduce variability due to illumination and contrast differences. The provided vessel masks were resized to match the corresponding image resolution and used only to compute a consistent retinal ROI (field-of-view) and suppress background content. These masks were not used as supervised targets, and no pixel-wise segmentation training or segmentation metrics are reported in this study. No handcrafted feature extraction was performed.

### 2.4 Model architecture for four-class disease recognition

For four-class disease recognition, we benchmarked two families of models:

1. Simple image-level classifiers based on ResNet50, DenseNet121, and EfficientNet-B0, initialized with ImageNet-pretrained weights. Each network was adapted to the four-class setting by replacing the final classification layer with a linear layer producing four logits for AMD, DR, glaucoma, and normal

2. DeepLabv3-based hybrid classifiers, in which DeepLabv3 (encoder + ASPP context head) models (with the same backbones) served as the primary architecture and were evaluated end-to-end for disease recognition. This hybrid setup enabled a controlled comparison between DeepLabv3–backbone hybrids and backbone-only simple classifiers, consistent with the hybrid-versus-simple comparisons presented in the Results.

In this manuscript, DeepLabv3 refers to a segmentation-style architecture whose defining component is the Atrous Spatial Pyramid Pooling (ASPP) context head. The ASPP head aggregates multi-scale context through parallel branches, typically including: (i) a 1 × 1 convolution branch, (ii) multiple 3 × 3 atrous (dilated) convolution branches with different dilation rates to capture features at different effective receptive fields, and (iii) an image-level context branch implemented via global average pooling followed by a 1 × 1 convolution. The outputs of these branches are concatenated and then fused via a 1 × 1 projection, yielding a context-enriched feature representation that is fed to the task head.

**FIGURE 2:**
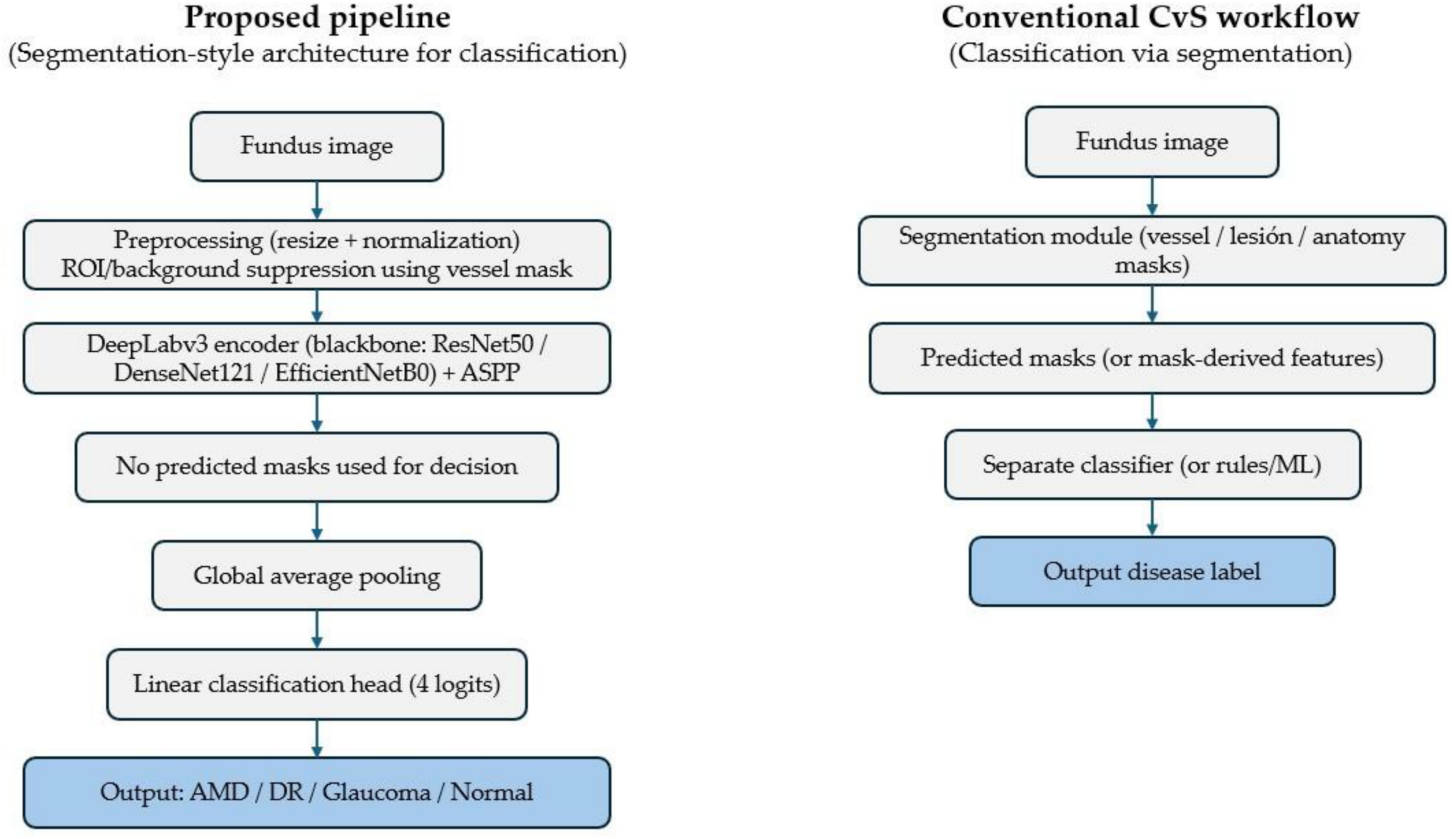
Schematic overview of the proposed pipeline vs. conventional classification-via-segmentation (CvS). The vessel mask is used only to define the retinal ROI and suppress background, while DeepLabv3 is repurposed end-to-end for image-level classification.

For the hybrid family, DeepLabv3–backbone networks were adapted for image-level prediction by replacing the task head with a four-class classification head and training end-to-end with cross-entropy. Therefore, there is no intermediate step in which segmentation outputs are converted into classification labels. Concretely, the original pixel-wise segmentation classifier is removed, and the ASPP-fused features are pooled to an image-level descriptor that is mapped to four logits (Figure 2).

In each hybrid variant, the DeepLabv3 module uses the ImageNet-pretrained backbone indicated in the model’s name (ResNet50, DenseNet121, or EfficientNet-B0), and the original pixel-wise segmentation classifier is replaced by a lightweight image-level head (global average pooling + linear layer). Accordingly, the comparison is DeepLabv3(backbone + ASPP context head) versus the corresponding standalone backbone classifier, not a stacked backbone (e.g., ResNet50 + DeepLabv3 + ResNet50).

### 2.5 Training procedure

All models were trained for 20 epochs using cross-entropy loss and optimized using AdamW under consistent hyperparameter settings. To quantify robustness, we performed five independent runs (*n* = 5) using different random seeds. For each run, a new stratified partition was generated, reserving 20% of the images as a held-out test set. The remaining 80% was used for training/validation under the same protocol. All reported metrics are computed on the 20% test set of each run and summarized as mean ± SD across the five runs. Within the 80% development set, we created a stratified validation subset (20%) used to monitor training and select the final checkpoint; the 20% test set remained strictly held out. Subject-level identifiers were not available in the public FIVES release; therefore, partitions were generated at the image level.

### 2.6 Evaluation metrics

Model performance was evaluated at the image level for multiclass screening and screening-oriented triage. For four-class disease recognition (AMD, DR, glaucoma, normal), we reported a set of global summary metrics—accuracy, sensitivity, specificity, and AUC—to enable direct comparison across model configurations. In the multiclass setting, sensitivity and specificity were computed using a one-versus-rest (OvR) definition for each class and then aggregated across classes to yield a single overall value (i.e., a macro-averaged summary). Likewise, discrimination was summarized using AUC computed with an OvR strategy and aggregated across the four classes to obtain an overall AUC, consistent with the model selection criterion.

To characterize error patterns beyond global metrics, we additionally examined class-level behavior (e.g., precision and recall) where reported in the corresponding results table/figures. Finally, to support a screening-oriented interpretation, we evaluated a binary triage formulation by collapsing outputs into referable disease (AMD ∪ DR ∪ glaucoma) versus normal, and computed sensitivity, specificity, PPV, and NPV from the resulting 2 × 2 confusion matrix, along with operational rates derived from these counts.

### 2.7 Statistical analysis

Results are reported as mean ± standard deviation across the five independent runs (*n* = 5). For comparative inference focused on discrimination, we complemented point estimates with t-based 95% confidence intervals for the mean AUC across runs and examined pairwise AUC differences between the selected model and comparators using Welch’s approach, i.e., Welch’s unequal-variance *t*-test with the Welch–Satterthwaite approximation for degrees of freedom applied to run-wise AUC estimates, applying multiplicity control where appropriate. Welch’s test is appropriate in this setting because the variability of run-wise AUC estimates may differ across models and we therefore do not assume equal variances (homoscedasticity); compared with the pooled-variance *t*-test, it provides a more robust comparison under heteroscedasticity, particularly for small sample sizes. This analysis was used to support robust claims about comparative performance beyond single-run outcomes and to quantify misclassification in the observed differences. Importantly, this misclassification reflects variability in the estimated performance metrics and their differences due to seed-controlled data partitioning (different 20% test sets) and training stochasticity across runs. We do not attempt to decompose epistemic versus aleatoric misclassification in this work. Thus, the reported run-to-run dispersion reflects both training stochasticity and variability due to evaluating on different held-out test samples; it is intended as a robustness check to partitioning, not as a substitute for external validation across centers or populations.

### 2.8 Implementation details

All experiments were implemented in Python using PyTorch and Torchvision, and training was performed in Google Colaboratory with GPU acceleration (NVIDIA Tesla T4). Pretrained ImageNet weights were used for encoder initialization when available. All reported results follow the five-run evaluation protocol described in section 2.5.

## 3 Results

### 3.1 Candidate model selection

As a first step toward a clinically implementable model for multiclass screening, we compared six configurations grouped into two families: (i) hybrid models based on DeepLabv3 + backbone (ResNet50, DenseNet121, and EfficientNet-B0) and (ii) plain backbone-based models used as classifiers. Performance is summarized in Table 2 using accuracy, sensitivity, specificity, and AUC. To support model comparison beyond point estimates, we report t-based 95% confidence intervals for mean AUC across runs and pairwise ΔAUC with 95% CIs for the selected model versus comparators.

**TABLE 2:** Performance of the six models evaluated on the multiclass screening task.

Family	Model	Accuracy	Sensitivity	Specificity	AUC
Hybrid	DeepLabv3-ResNet50	0.924 ± 0.025	0.851 ± 0.049	0.949 ± 0.017	0.969 ± 0.021
Hybrid	DeepLabv3-DenseNet121	0.939 ± 0.017	0.878 ± 0.034	0.960 ± 0.011	0.979 ± 0.009
Hybrid	DeepLabv3-EfficientNet-B0	0.936 ± 0.019	0.874 ± 0.038	0.957 ± 0.012	0.976 ± 0.013
Simple	ResNet50	0.941 ± 0.021	0.879 ± 0.041	0.961 ± 0.014	0.920 ± 0.027
Simple	DenseNet121	0.929 ± 0.016	0.863 ± 0.031	0.952 ± 0.011	0.908 ± 0.021
Simple	EfficientNet-B0	0.937 ± 0.012	0.876 ± 0.024	0.958 ± 0.008	0.917 ± 0.016

Mean ± standard deviation across five independent runs (*n* = 5) are reported for accuracy, sensitivity, specificity, and AUC.

**FIGURE 3:**
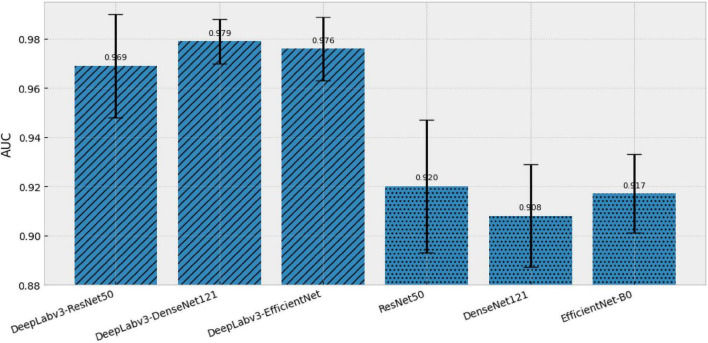
AUC for the six models evaluated in the multiclass screening task. Bars show the mean AUC, and error bars indicate the standard deviation across *n* = 5 runs. A visual distinction is made between hybrid and single (non-hybrid) models.

In terms of accuracy, all models achieved high and closely clustered values (0.924–0.941), with sensitivities ranging from 0.851 to 0.879 and specificities from 0.949 to 0.961. However, the most relevant criterion for future deployment, given its threshold-independence and its direct relationship with the quality of the probabilistic score, was AUC, where a clear separation between families was observed: the hybrid models achieved consistently high AUCs (0.969–0.979), whereas the plain models remained in a lower range (0.908–0.920). This difference is directly visualized in Figure 3, which shows the mean AUC per model with error bars, highlighting the systematic advantage of the hybrid family in discriminative capability.

**FIGURE 4:**
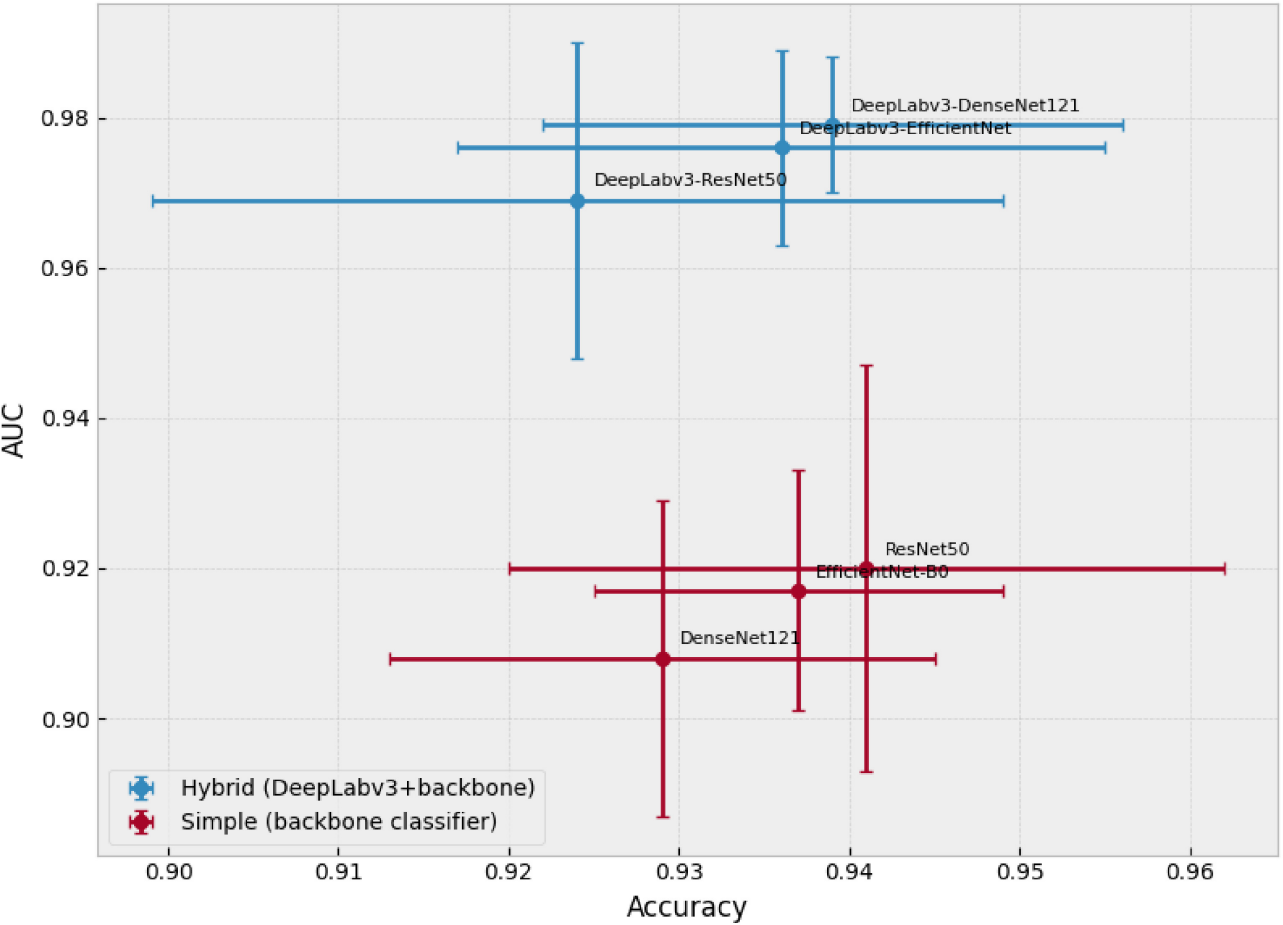
Trade-off between accuracy and AUC for all models. Each point represents the mean performance per model; error bars correspond to the standard deviation (*n* = 5) for accuracy (X-axis) and AUC (Y-axis). This representation allows simultaneous comparison of threshold-dependent accuracy and overall discriminative ability.

Among the evaluated models, DeepLabv3–DenseNet121 achieved the best AUC (0.979 ± 0.009), along with competitive performance across the other metrics. In contrast, the highest accuracy corresponded to ResNet50 (0.941 ± 0.021), but with a considerably lower AUC (0.920 ± 0.027), suggesting that comparisons based solely on accuracy can mask meaningful differences in the probabilistic ranking of cases. This idea is reinforced in Figure 4, where the accuracy–AUC plane shows that the hybrid models shift toward the higher-AUC region with bounded variability, whereas the single models remain clustered at lower AUC values.

Finally, to isolate the effect of the hybrid approach while controlling for the backbone, Supplementary Figure 1 shows the backbone-specific gain as Δ(Hybrid − Simple). The pattern is consistent: the hybrid improves AUC relative to its simple counterpart across all three backbones (approximate gains of +0.049 to +0.071), whereas changes in accuracy, sensitivity, and specificity are modest and may be positive or negative depending on the backbone.

### 3.2 Class-wise behavior of the best model

**FIGURE 5:**
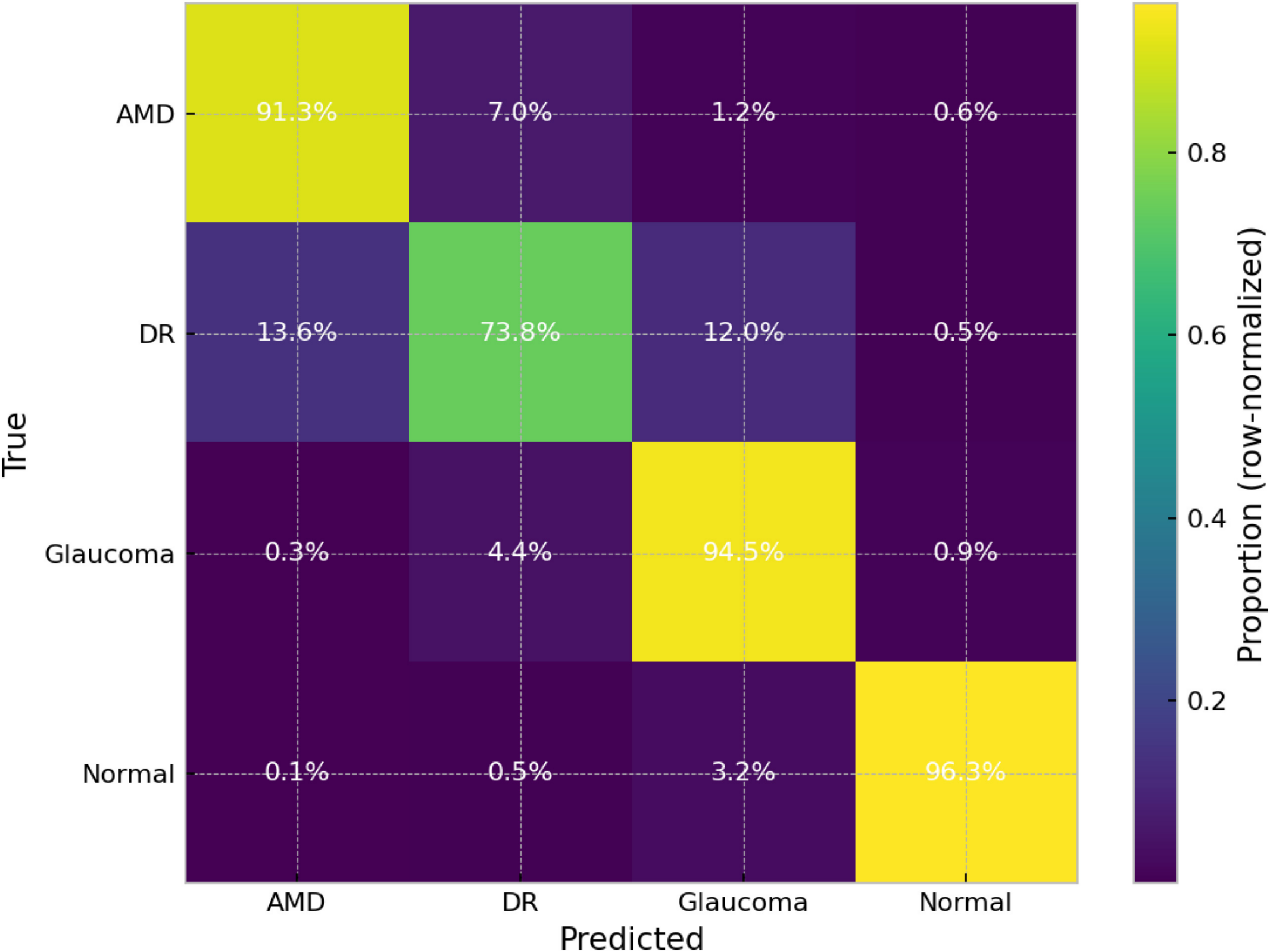
Mean confusion matrix of the DeepLabv3–DenseNet121 model (*n* = 5), row-normalized; values in % relative to the true class.

After selecting DeepLabv3–DenseNet121 as the candidate model, we characterized its pathology-specific behavior using five independent runs. For a clinically oriented interpretation, we combined the row-normalized confusion matrix (Figure 5) with the per-class metrics (Table 3).

**TABLE 3:** Class-wise metrics (AMD, DR, Glaucoma, Normal) of the DeepLabv3–DenseNet121 model across 5 independent runs: mean ± SD of precision, sensitivity, F1-score, and OvR specificity.

Class	Precision	Recall/sensitivity	F1-score	Specificity
AMD	0.871 ± 0.060	0.913 ± 0.034	0.890 ± 0.026	0.955 ± 0.027
DR	0.868 ± 0.038	0.738 ± 0.117	0.793 ± 0.072	0.963 ± 0.014
Glaucoma	0.858 ± 0.093	0.945 ± 0.030	0.896 ± 0.048	0.946 ± 0.035
Normal	0.980 ± 0.032	0.963 ± 0.034	0.970 ± 0.014	0.993 ± 0.011
Macro avg	0.894 ± 0.025	0.890 ± 0.029	0.887 ± 0.032	0.964 ± 0.009

Macro avg = unweighted average.

Overall, the model shows high and consistent performance on the Normal class (Precision 0.980 ± 0.032; Sensitivity 0.963 ± 0.034; F1 0.970 ± 0.014; OvR Specificity 0.993 ± 0.011), and solid results for Glaucoma (Sensitivity 0.945 ± 0.030; F1 0.896 ± 0.048) and AMD (Sensitivity 0.913 ± 0.034; F1 0.890 ± 0.026). The most challenging class is DR, with the lowest sensitivity (0.738 ± 0.117) and the highest variability across runs (Table 3).

Figure 5 reveals the dominant confusion patterns: AMD is correctly classified in 91.3% of cases, with the main deviation toward DR (7.0%); Glaucoma reaches 94.5%, with preferential confusion toward DR (4.4%); and Normal achieves 96.3%, with the primary error toward Glaucoma (3.2%). OvR specificity remains high across all classes (0.946–0.993), indicating a low tendency to overdiagnose any one category against the rest.

The critical class is DR, with a mean sensitivity of 73.8% and errors concentrated in DR→AMD (13.6%) and DR→Glaucoma (12.0%), while DR→Normal is marginal (0.5%). In practical terms, the model rarely confuses DR with a healthy eye, but it does interchange DR with other pathologies, which would still preserve referral in a multiclass triage setting. Overall, performance remains well balanced on average (Macro avg: Precision 0.894 ± 0.025; Sensitivity 0.890 ± 0.029; F1 0.887 ± 0.032; Specificity 0.964 ± 0.009), with DR as the main bottleneck.

This DR bottleneck is plausibly explained by the nature of DR cues: compared with glaucoma (often expressed through more global optic-disc appearance), DR frequently manifests as small, localized, and low-contrast lesions (e.g., microaneurysms, small hemorrhages, exudates) that can be spatially sparse. Such fine-grained signals may be partially attenuated by resizing to 224 × 224 and by variability in illumination or imaging artifacts, increasing intra-class variability and confusability with other pathologies. Because the four-class FIVES dataset is class-balanced in our multiclass setting, class imbalance is unlikely to be the primary driver of the lower DR sensitivity; instead, the observed errors are consistent with subtle lesion visibility and underlying within-class heterogeneity.

### 3.3 Statistical robustness and model comparison

**FIGURE 6:**
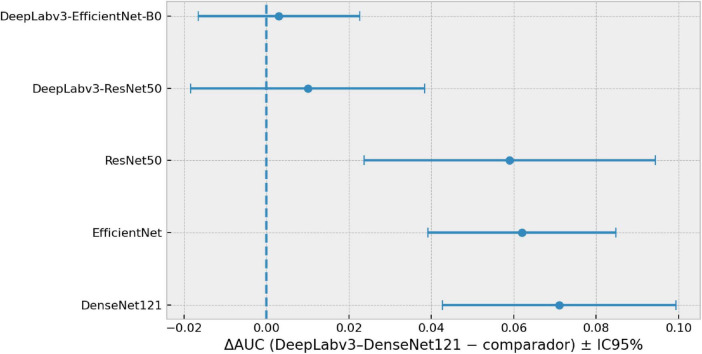
Pairwise AUC differences ΔAUC (selected model - comparator) computed from run-wise AUC estimates, with t-based 95% confidence intervals (intervals crossing zero indicate differences compatible with zero).

To determine whether the advantage of the selected model (DeepLabv3–DenseNet121) is robust and not the result of a favorable test split, we analyzed the variability across the five independent runs and compared its performance against the remaining configurations. Figure 6 shows the AUC difference (ΔAUC) between the selected model and each comparator, also with 95% CIs.

Hybrid models concentrate the highest AUC values, and DeepLabv3–DenseNet121 lies at the upper end of the performance range while also exhibiting reduced uncertainty (lower run-to-run variability), consistent with greater stability across runs. This stability is relevant because it suggests that the candidate’s performance is reproducible under seed-controlled data partitioning.

Figure 6 reinforces this conclusion by plotting ΔAUC (selected model − comparator). The selected model shows a clear advantage over the single models, with positive differences and 95% CIs that do not cross zero, indicating consistent improvements in discriminative ability. In contrast, compared with other hybrid models, the differences are small and the 95% CIs are compatible with zero, suggesting that performance among hybrids is comparable and that choosing DeepLabv3–DenseNet121 is mainly justified by a combination of high AUC and lower variability, rather than by a pronounced superiority over all hybrid models.

Overall, this evidence indicates that the selected model not only maximizes the average AUC but also identifies a model with stable and reproducible behavior, an essential requirement when the goal is to move toward clinical use, where consistency of performance is as important as the mean achieved.

### 3.4 Run-to-run stability and variation-sensitive classes

Beyond average performance, we assessed the stability of the selected model across its five runs to identify which classes and which confusions are most sensitive to run-to-run variability. To this end, we analyzed (i) per-class sensitivity in each run and (ii) the standard deviation of the row-normalized confusion matrix, which quantifies the dispersion of misclassification patterns across runs.

**FIGURE 7:**
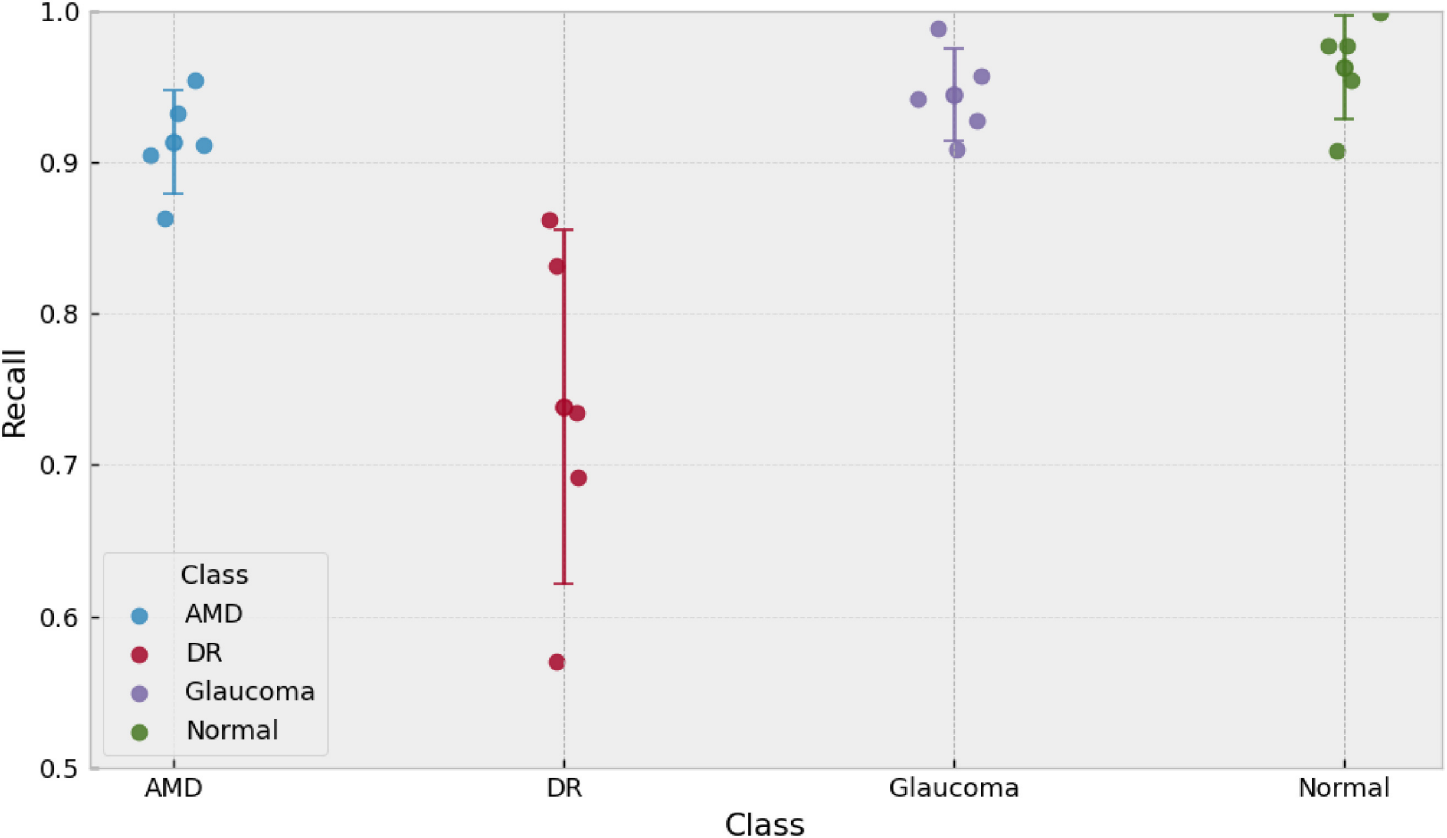
Per-class sensitivity (recall) of the selected model across five independent runs (*n* = 5). Points denote run-wise sensitivities and error bars indicate the mean ± standard deviation for each class. Runs are independent and no across-run relationship is implied.

Figure 7 shows that Normal, Glaucoma, and AMD maintain relatively consistent sensitivities across runs, whereas DR exhibits the largest fluctuations, confirming it as the class most dependent on training and split variability. This observation is consistent with the higher variability already reported in section 3.2 and suggests that DR detection may be more vulnerable to subtle domain shifts (image quality, illumination, lesion distribution, or artifacts).

**FIGURE 8:**
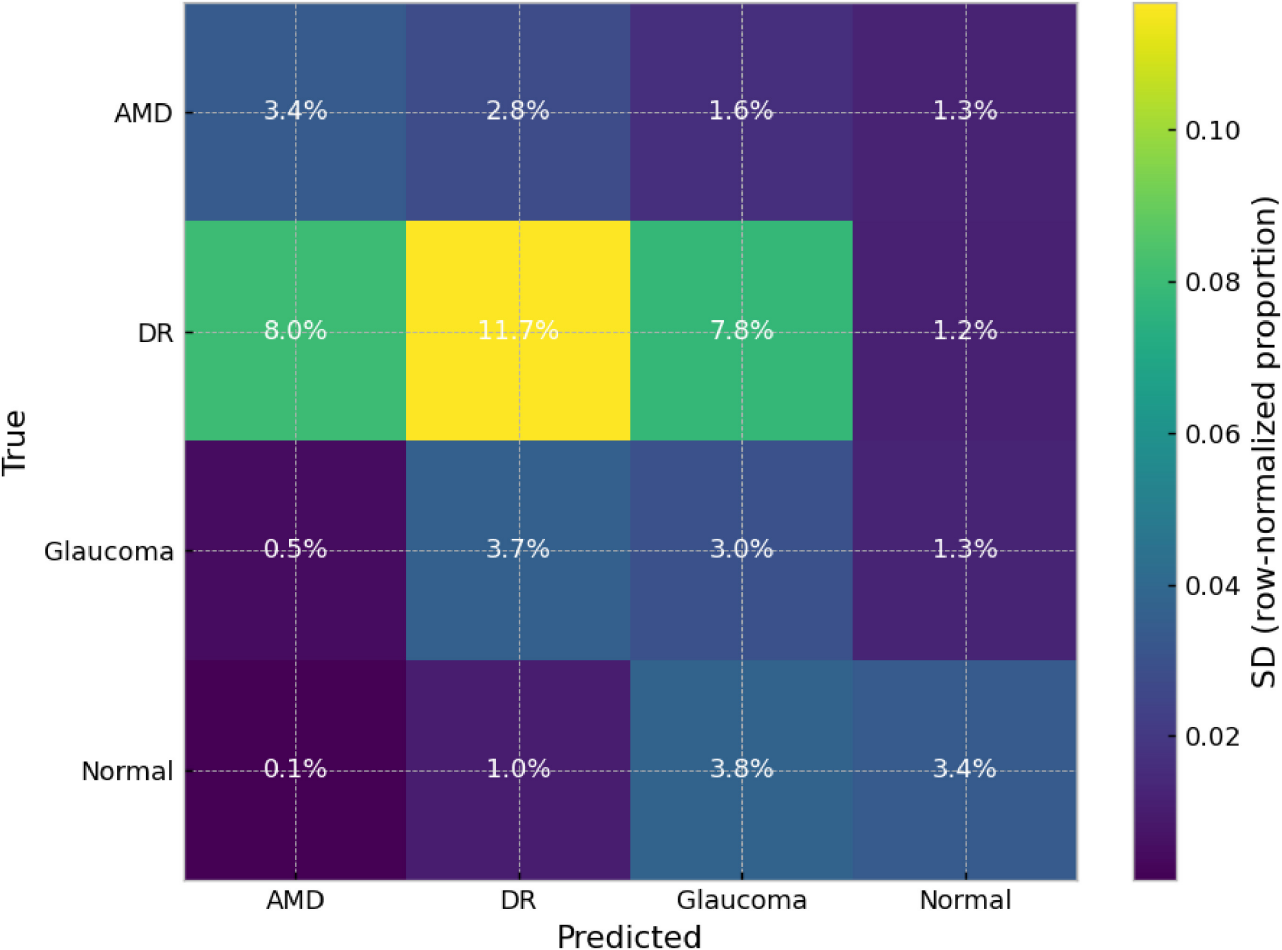
Standard deviation (SD) of the row-normalized confusion matrix for DeepLabv3–DenseNet121 across five independent runs (*n* = 5). Values indicate the SD (in %) of the per-row proportions, highlighting which misclassification patterns are most variable across runs.

Figure 8 helps pinpoint where this variability is concentrated: cells associated with confusions involving DR (e.g., DR→AMD and DR→Glaucoma) show greater relative dispersion than the rest, whereas the characteristic correct and incorrect predictions for the Normal class remain more stable. Overall, these results indicate that the model’s global robustness is good, but that run-to-run misclassification variability is concentrated around the decision boundaries separating DR from AMD/Glaucoma. This, in turn, justifies incorporating, in later sections, calibration/thresholding mechanisms or human-review criteria specifically targeted at this confusion region. We note that this section characterizes misclassification variability across runs rather than formal predictive uncertainty quantification.

### 3.5 Triage-oriented clinical evaluation and operational robustness: “referable” vs. “normal”

In addition to the multiclass analysis, we evaluated the selected model under a clinical triage scenario by collapsing the four categories into a binary problem: referable (AMD ∪ DR ∪ Glaucoma) versus normal. This approach is particularly relevant in screening settings, where the primary goal is often to maximize disease detection (minimizing false negatives) while keeping referral workload manageable.

**FIGURE 9:**
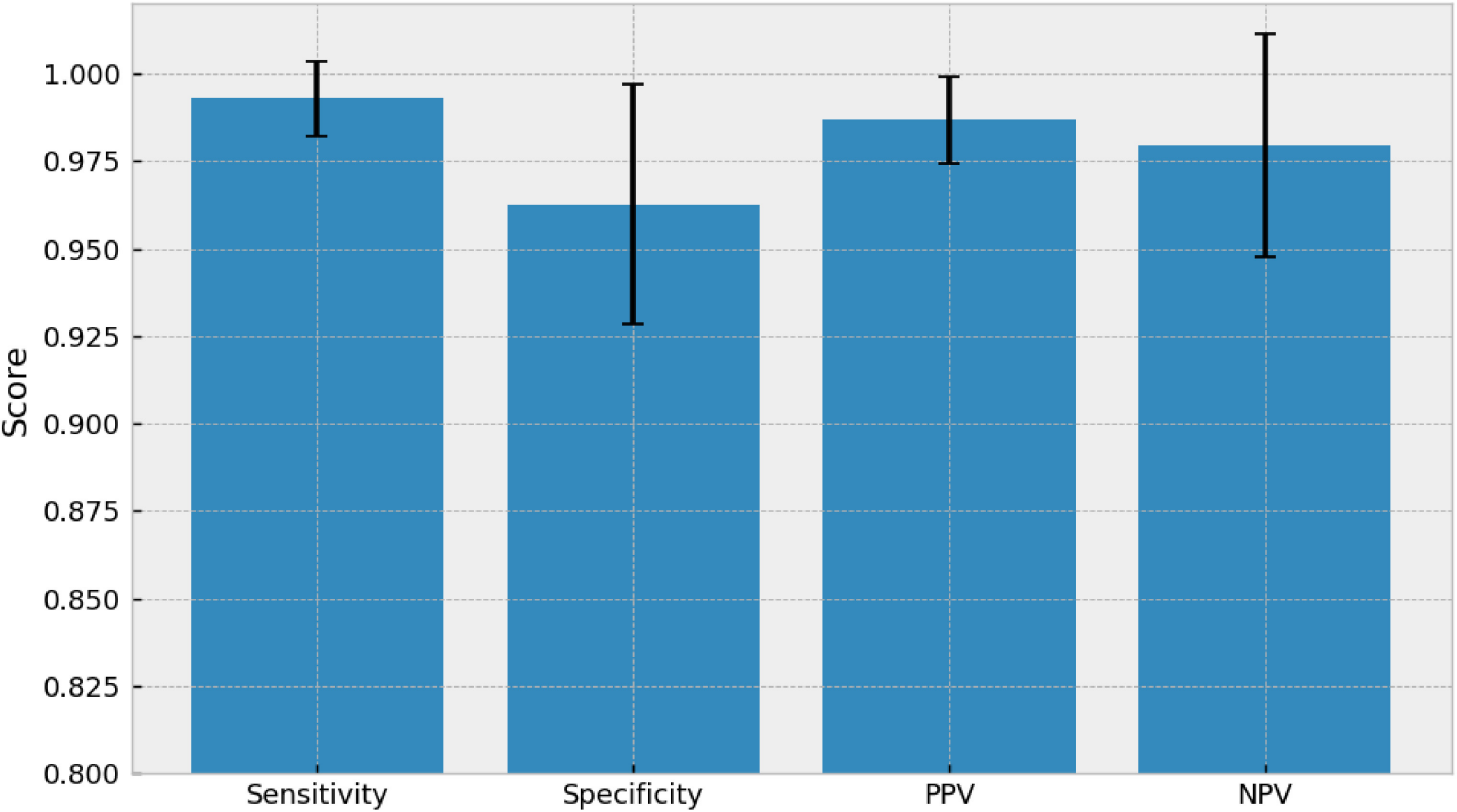
Binary triage performance (referable = AMD ∪ DR ∪ Glaucoma vs. normal) of the DeepLabv3–DenseNet121 model across five independent runs (*n* = 5). Sensitivity, specificity, PPV, and NPV are reported as mean ± SD.

Based on the confusion matrices from five independent runs, the binary triage achieved high performance (Figure 9): sensitivity 0.993 ± 0.011, specificity 0.963 ± 0.034, PPV 0.987 ± 0.013, and NPV 0.980 ± 0.032. Overall, these values indicate that the system consistently identifies most referable cases, with a low likelihood of classifying an eye with pathology as normal, while maintaining an adequate level of discrimination against normal cases.

**FIGURE 10:**
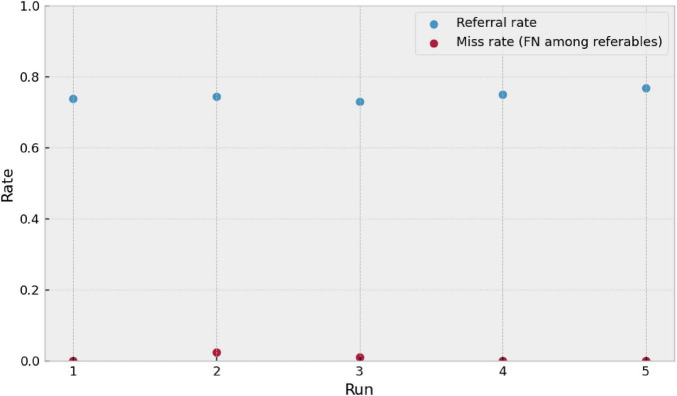
Operational triage stability across five independent runs (*n* = 5; distinct 20% test splits). Blue dots show the referral rate (fraction predicted as referable), and red dots show the miss rate among referables [FN/(TP+FN)]. Runs are independent and no across-run relationship is implied.

From an operational perspective, the referral rate remained within a narrow range across runs (∼0.73–0.77) (Figure 10), suggesting stable behavior in terms of clinical workload under the evaluated dataset distribution. In parallel, the miss rate for referable cases [miss rate, FN/(TP+FN)] was low across all runs, though not identical, indicating that the residual risk is concentrated in borderline decisions. Runs are independent and the plot does not imply any across-run relationship.

Finally, it should be noted that both the referral rate and the predictive values depend on the prevalence and composition of the evaluation set. Therefore, a real-world deployment would require external validation and, where appropriate, threshold adjustment and/or calibration; nevertheless, these results provide an operational perspective that complements multiclass performance by quantifying the trade-off between clinical safety (sensitivity), workload (referral rate), and run-to-runstability.

### 3.6 Operational synthesis and deployment considerations

**FIGURE 11:**
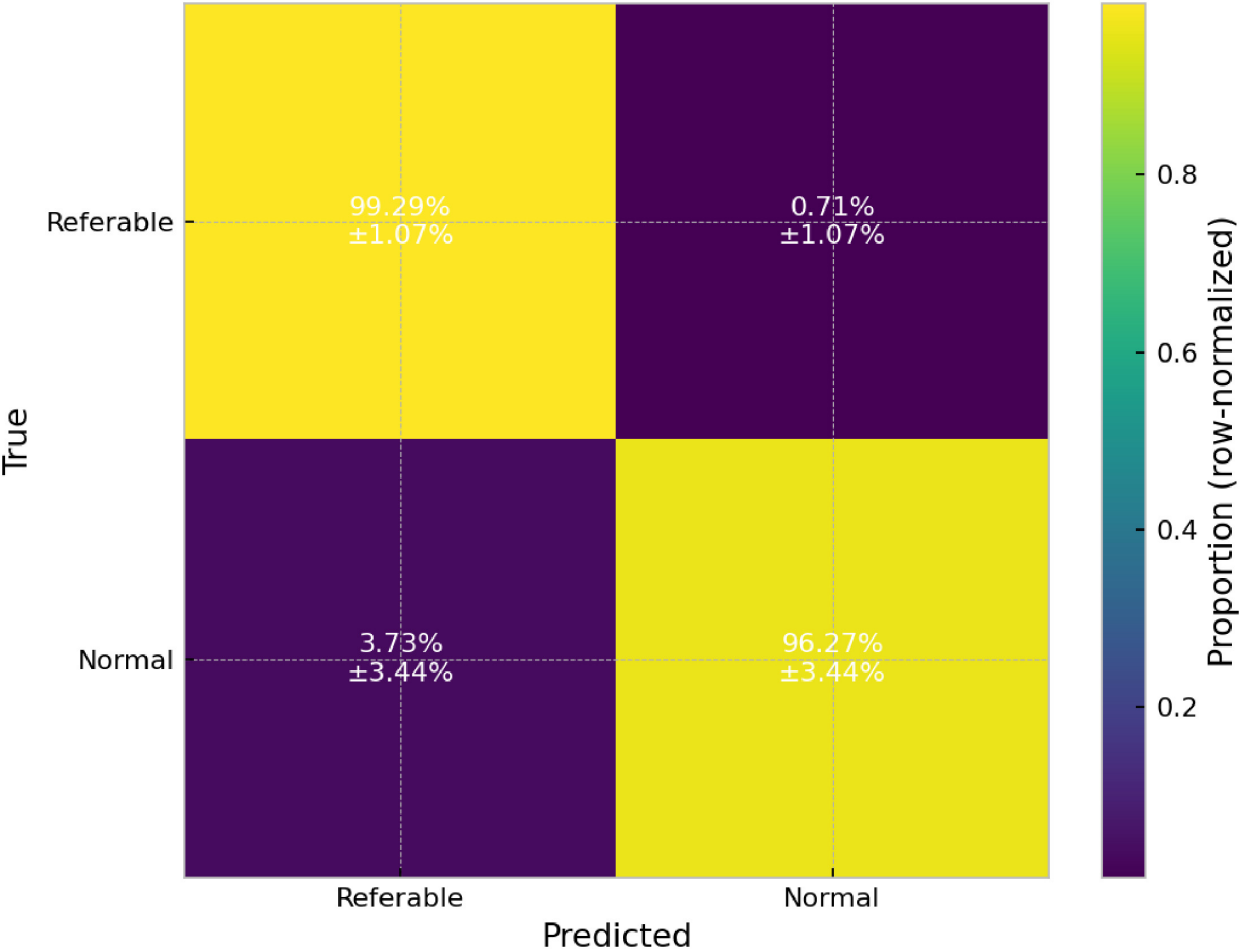
Row-normalized binary triage (referable vs. normal) confusion matrix for the DeepLabv3–DenseNet121 model, showing mean ± SD (%) across five independent runs.

Based on the multiclass results and the triage analysis, we propose an operational screening policy: refer cases predicted as AMD/DR/Glaucoma and treat Normal as non-referable, while adopting a conservative stance in the DR–AMD–Glaucoma confusion region. To communicate the “safety vs. workload” trade-off more directly, we summarize the five runs using a row-normalized binary confusion matrix (mean ± SD) (Figure 11). This representation captures the probability of (i) correctly classifying a referable case as referable (sensitivity), (ii) missing a referable case as Normal (miss rate), and (iii) falsely referring a Normal case (false positives), providing a compact operational view of screening behavior.

## 4 Discussion

This study evaluated six configurations (hybrid and single) for classifying ocular pathologies (AMD, DR, Glaucoma, and Normal) and selected DeepLabv3–DenseNet121 as the candidate model due to its combination of high discriminative power and run-to-runstability. The results support three central messages: (i) AUC-based selection is consistent with a diagnostic objective, as it better captures overall discriminative ability than threshold-dependent metrics such as accuracy; (ii) performance is heterogeneous across classes, with DR as the main bottleneck; and (iii) when the task is reframed as “referable vs. normal” triage, the system maintains high sensitivity with a bounded referral burden, naturally aligning with a potential clinical screening workflow. In scientific terms, consistent gains across models/backbones indicate that segmentation-style multi-scale context aggregation can be beneficial for image-level fundus screening under the studied protocol, whereas inconsistent or small differences should be interpreted as within-dataset effects that warrant external validation before clinical claims.

The class-wise characterization shows that Normal is the most separable and stable category, whereas the greatest misclassification concentrates within the pathological block, particularly around DR. The confusion matrix indicates that DR tends to be exchanged with AMD and Glaucoma, a clinically plausible pattern given phenotypic variability, image quality, and overlap of retinal signals across entities (especially in early stages or in the presence of artifacts). In addition, DR often relies on subtle, lesion-level evidence that may be harder to preserve under aggressive downsampling than the more global cues typically leveraged for glaucoma, which may further contribute to DR being the lowest-performing class in our setting. In contrast, the fact that DR is rarely confused with Normal suggests that the model fails more by relabeling within pathology than by “missing” pathology as healthy, which is favorable from a multiclass screening perspective. Nonetheless, the run-to-run variability associated with DR indicates that its detection may be more sensitive to subtle domain and distribution shifts, reinforcing the need for conservative strategies when the system operates near that boundary.

Model comparisons further support the selected choice: although some single configurations may come close in accuracy, the AUC criterion better discriminates among alternatives, showing that single models lag in global discriminative capability. Within the hybrid family, AUC differences among candidates are small, suggesting a shared performance ceiling, in that setting, DeepLabv3-DenseNet121 stands out for its balance of high AUC and lower dispersion an attribute that is critical when reproducibility and predictable behavior across runs are desired.

Because our hybrid models use a single backbone encoder inside DeepLabv3 (section 2.4) and do not place an additional deep CNN on top, the observed AUC gains are unlikely to be driven by a simple stacking of CNN backbones. Instead, they more plausibly reflect DeepLabv3’s ASPP-based multi-scale context aggregation and segmentation-style feature extraction; nevertheless, DeepLabv3 introduces additional parameters beyond the plain backbone (e.g., ASPP and related layers), so part of the improvement may also be attributable to this added capacity.

The binary triage analysis provides a directly actionable operational reading: by collapsing classes into referable (AMD/DR/Glaucoma) vs. normal, the system preserves high sensitivity and a stable referral pattern. This interpretation is consistent with the typical goal of screening, where minimizing false negatives is prioritized even at the cost of some over-referral. However, both the referral rate and PPV/NPV depend on the true prevalence in the deployment setting; therefore, although the observed performance is promising, it should not be extrapolated without external validation across different populations and devices. Practically, the results suggest a conservative policy: refer whenever the system predicts AMD/DR/Glaucoma and consider “no referral” only for Normal predictions, concentrating risk control in the DR–AMD–Glaucoma region (e.g., human review when a case lies near that boundary or when instability is detected).

This work has relevant limitations: (i) generalization may be affected by domain shift (device, center, protocol, prevalence); (ii) the operational analysis is derived from confusion matrices and, without per-sample probabilities, it is not possible to fully assess calibration or optimize thresholds; (iii) visual explainability (e.g., Grad-CAM) was not included, limiting auditing of the regions driving decisions and detection of potential shortcuts; (iv) although our repeated runs use different 20% test splits, they do not capture multi-center population heterogeneity, label noise, or distribution shift; external validation is still required for real-world reliability; and (v) classes aggregate clinical heterogeneity and unlabeled disease severity, which may especially penalize DR by increasing intra-class variability. Because severity-stage annotations are not available in FIVES, we cannot stratify performance by DR severity in this study. As avenues for improvement, we recommend external validation, subgroup analyses (image quality, severity), calibration and risk-based referral rules, and the incorporation of explainability for clinical auditing. Overall, the results support DeepLabv3–DenseNet121 as a strong candidate to advance toward screening scenarios, with the caveat that the DR–AMD–Glaucoma confusion region should be the primary focus of risk control and additional validation prior to any clinical deployment.

## 5 Conclusion

This study compared six model configurations for classifying ocular pathologies (AMD, DR, Glaucoma, and Normal) and selected DeepLabv3–DenseNet121 as the candidate due to its high AUC and run-to-run stability. The class-wise analysis showed robust performance for Normal, Glaucoma, and AMD, while DR was identified as the main bottleneck, with predominant confusions toward AMD and Glaucoma. Even so, the model rarely confuses DR with Normal, which supports its use in screening scenarios.

From a clinical application perspective, when the task collapsed into binary triage (referable vs. normal), the system maintained high sensitivity and a relatively stable referral burden across the five runs, providing an operationally meaningful and directly interpretable output for clinical workflows. Overall, the results support DeepLabv3–DenseNet121 as a solid basis to move toward external validation and deployment-oriented optimization, prioritizing risk control in the DR–AMD–Glaucoma confusion region and, in future work, complementing with calibration and visual explainability (e.g., Grad-CAM) to strengthen clinical trust.

## Data Availability

The data analyzed in this study are publicly available through the FIVES dataset and can be accessed via the article cited as reference (25). Further inquiries can be directed to the corresponding author.
