# Supplementary material for: Robust benchmarking of DeepLabv3 hybrid models for multiclass fundus screening and referable-disease triage on the FIVES dataset
Source: Front Med (Lausanne). 2026 Mar 31;13:1771083. doi: 10.3389/fmed.2026.1771083 (PMC13076344; doi:10.3389/fmed.2026.1771083)
Supplement: Supplementary file 1 [file Table_1.docx]

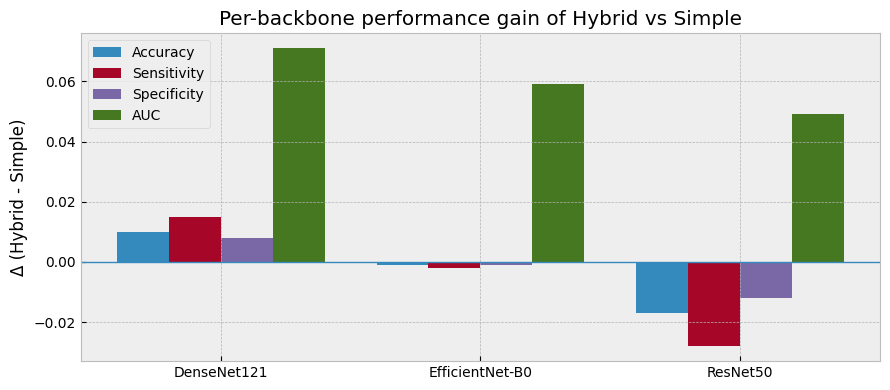
Supplementary Material

Figure S1. Improvement of the hybrid approach over the simple one, expressed as Δ(Hybrid − Simple) for accuracy, sensitivity, specificity, and AUC. Positive values indicate that the hybrid model outperforms its simple counterpart when using the same backbone.
